# Second primary cancers of the breast: incidence and risk factors.

**DOI:** 10.1038/bjc.1984.12

**Published:** 1984-01

**Authors:** T. G. Hislop, J. M. Elwood, A. J. Coldman, J. J. Spinelli, A. J. Worth, L. G. Ellison

## Abstract

Between 1946 and 1976 over 9,000 women with breast cancer were seen within one year of diagnosis at the A. Maxwell Evans Clinic (AMEC) in Vancouver, British Columbia. By 1978, 275 had a subsequent diagnosis of a second primary in the contralateral breast: 100 were diagnosed within 1 year, and 175 after 1 year of the first primary. Two separate comparison groups of AMEC patients with unilateral breast cancer were selected to identify risk factors for bilateral breast cancer and to determine the incidence. The average annual incidence rates for a second primary in the contralateral breast were 5.0, 4.1 and 3.0 per 1,000 women for women less than 45 years, 45-54 years, and over 55 years of age at diagnosis of first primary breast cancer, respectively. These rates remained stable for at least 15 years after the diagnosis of the first primary. Two risk factors were found for bilateral cancer within 1 year of the first primary, histologic diagnosis of lobular carcinoma and absence of pathologic involvement of axillary nodes; one risk factor was found for bilateral breast cancer after 1 year of the first primary, family history of breast cancer.


Br. J. Cancer (1984), **49**, 79–85

### Second primary cancers of the breast: Incidence and risk factors

T.G. Hislop[1], J.M. Elwood[4], A.J. Coldman[1], J.J. Spinelli[1], A.J. Worth[3] & L.G. Ellison[2]

[1]*Division of Epidemiology & Biometry;* [2]*Division of Clinic Physicians;* [3]*Division of Pathology; Cancer Control Agency of British Columbia, Canada and* [4]*Department of Community Health, University of Nottingham, U.K.*

**Summary** Between 1946 and 1976 over 9,000 women with breast cancer were seen within one year of diagnosis at the A. Maxwell Evans Clinic (AMEC) in Vancouver, British Columbia. By 1978, 275 had a subsequent diagnosis of a second primary in the contralateral breast: 100 were diagnosed within 1 year, and 175 after 1 year of the first primary. Two separate comparison groups of AMEC patients with unilateral breast cancer were selected to identify risk factors for bilateral breast cancer and to determine the incidence. The average annual incidence rates for a second primary in the contralateral breast were 5.0, 4.1 and 3.0 per 1,000 women for women less than 45 years, 45–54 years, and over 55 years of age at diagnosis of first primary breast cancer, respectively. These rates remained stable for at least 15 years after the diagnosis of the first primary. Two risk factors were found for bilateral cancer within 1 year of the first primary, histologic diagnosis of lobular carcinoma and absence of pathologic involvement of axillary nodes; one risk factor was found for bilateral breast cancer after 1 year of the first primary, family history of breast cancer.

With longer survival rates from breast cancer the risk increases that a woman will develop a second breast malignancy. Information is needed about this likelihood and the characteristics of women at high risk, especially when considering such issues as prophylactic contralateral mastectomy. This study was undertaken to determine factors which influence the incidence of second primary tumours of the contralateral breast.

### Method

Over 9,000 women had their first histologically confirmed primary breast cancer diagnosed between 1946 and 1976 and were registered for treatment within one year of diagnosis at the A. Maxwell Evans Clinic (AMEC) in Vancouver, British Columbia. Two hundred and seventy-five of these women subsequently had an invasive second primary diagnosed in the contralateral breast prior to November 1978 and they comprised the bilateral cancer group ("cases"). Two comparison groups ("controls") were selected from the remaining women with unilateral breast cancer: one comparison group, the "5% sample", was a randon 5% sample of these women; the second comparison group, the "matched sample", consisted of 275 women individually matched with the cases by age ($\pm 2$ years), year of diagnosis of the first primary ($\pm 1$ year) and survival (required to be greater than the elapsed time between the diagnosis of the first and second primary breast cancers in the case).

The medical records of the women in the bilateral cancer group and two comparison groups were reviewed in 1978, restricting the observations on risk factors to those available at the time of referral for the first breast primary. None were lost to follow-up. Information was collected on recognised risk factors for both unilateral and bilateral breast cancer and clinical details of the first primary breast cancer. A case-control study design was then used to identify the risk factors associated with bilateral breast cancer. All women with bilateral breast cancer were compared to the matched sample to estimate the relative risk for the various study factors. This was done separately for women with a second primary diagnosed within 1 year ("synchronous cases") and after 1 year ("asynchronous cases") of the diagnosis of the first primary. Analysis was made preserving the matching using classical matched-pair methods (Breslow & Day, 1980).

The incidence of second primary tumours was obtained using a life table method where death or loss to follow up were considered as censored observations (Peto *et al.*, 1977). The study group consisted of the asynchronous cases and the 5% sample. The incidence was calculated separately for women of ages $\leqslant 44$ years, 45–54 years and $\geqslant 55$ years at the time of diagnosis of the first primary in order to see if age at diagnosis affected one's risk

Correspondence: T.G. Hislop

Received 15 June 1983; accepted 23 September 1983.

© The Macmillan Press Ltd., 1984

for a second primary. Age specific incidence rates were also calculated for significant risk factors in asynchronous cases. Differences in incidence were tested for significance using the logrank statistic and its multivariate generalization (Peto *et al.*, 1977).

### Results

Of the 275 women with bilateral breast cancer, 100 were synchronous, 60 being diagnosed within one month, and 175 were asynchronous. Differences resulting from matching between the control groups are shown in Table I. Women in the 5% sample tended to be older, with more advanced disease, and diagnosed in more recent years than women in the matched sample.

### *Risk factors for bilateral breast cancer*

The length of the time interval between diagnosing the first and second primaries influenced the type of risk factors for bilateral breast cancer. The risk of diagnosing a second primary within one year of the first primary was significantly elevated for women with a lobular carcinoma and for women with no pathologically determined involvement of axillary nodes. Prior non-contraceptive oestrogen use was of borderline significance (Table II). These three risk factors were independent as the odds ratio for each remained essentially unchanged after controlling for the other two factors.

The risk of diagnosing a second primary after one year of the first primary was significantly elevated for women who reported a history of breast cancer in a first degree relative (Table III). This elevated risk was uniform across different age groups at first diagnosis.

None of the other factors which were examined significantly altered the relative risk of bilateral breast cancer, even after controlling for the significant risk factors. These factors included age at first birth, parity, age at menarche, prior oral contraceptive use, weight, history of other diseases (cancer, benign breast disease, hypertension, thyroid disease), type of early signs or symptoms, multifocal tumours, histologic grade, delay time to diagnosis and to treatment, and location of the tumour.

### *Incidence of bilateral breast cancer*

Figure 1 indicates the observed incidence of a second primary in the contralateral breast for 3 groups, women of ages $<45$ years, 45–54 years, and $\geqslant 55$ years at the time of diagnosis of the first primary. These rates were restricted to women with at least a one year interval between the diagnosis of the first and second primaries; hence the number of women at risk in the 5% sample was reduced to 376 which gives an estimate of 7,800 for all women at risk in this study population.

There is a non-significant trend of decreasing incidence with increasing age and a clear linear relationship between cumulative incidence and years

**Table I** Distribution of characteristics related to matching criteria by study group

| *Characteristic (at diagnosis of the first primary)* | *Bilateral breast cancer ("cases")* | *Unilateral breast cancer ("controls")* | |
|---|---|---|---|
| | | *Matched sample* | *5% sample* |
| Total number | 275 | 275 | 438 |
| Age (mean years) | 53.0 | 53.0 | 56.2 |
| Year of diagnosis (%) | | | |
| 1940s | 4 | 4 | 3 |
| 1950s | 24 | 24 | 19 |
| 1960s | 43 | 43 | 36 |
| 1970s | 29 | 29 | 42 |
| Clinical stage (%) | | | |
| I | 61 | 61 | 51 |
| II | 23 | 23 | 22 |
| III | 12 | 9 | 11 |
| IV | 4 | 7 | 15 |
| Presence of Invasion (%) | | | |
| In situ only | 4 | 3 | 2 |
| Invasive | 96 | 97 | 98 |

**Table II** Risk factors for synchronous bilateral breast cancer

| *Factor (refers to first primary)* | *Percent with factor*[a] *Cases* | *Matched controls* | *Matched odds ratio* | *95% C.I.* | *P value* |
|---|---|---|---|---|---|
| Breast cancer in mother or sister | 17% | 12% | 1.6 | 0.6– 4.1 | 0.41 |
| Lobular carcinoma | 18% | 6% | 4.3 | 1.2–23.6 | 0.02 |
| Pathologic axillary nodes | 34% | 48% | 0.5 | 0.3– 1.0 | 0.05 |
| Prior oestrogen use | 20% | 10% | 2.4 | 1.0– 6.9 | 0.06 |
| Late age at first birth[b] | 68% | 53% | 2.7 | 0.6–15.5 | 0.23 |
| Prior benign breast disease | 12% | 10% | 1.2 | 0.5– 3.3 | 0.82 |
| Multifocal tumours within same breast | 7% | 2% | 3.5 | 0.7–34.5 | 0.18 |

[a]Percent of all cases, and matched controls, with factor; not restricted to discordant pairs.
[b]Late age at first birth dichotomized at <25 years and ⩾25 years and restricted to women who have given birth.

**Table III** Risk factors for asynchronous bilateral breast cancer

| *Factor (refers to first primary)* | *Percent with factor*[a] *Cases* | *Matched controls* | *Matched odds ratio* | *95% C.I.* | *P value* |
|---|---|---|---|---|---|
| Breast cancer in mother or sister | 19% | 7% | 3.1 | 1.5– 7.1 | 0.001 |
| Lobular carcinoma | 8% | 8% | 1.0 | 0.4– 2.5 | 1.00 |
| Pathologic axillary nodes | 43% | 43% | 1.0 | 0.7– 1.5 | 1.00 |
| Prior oestrogen use | 9% | 7% | 1.3 | 0.5– 3.1 | 0.69 |
| Late age at first birth[b] | 63% | 54% | 1.5 | 0.7– 3.4 | 0.30 |
| Prior benign breast disease | 9% | 17% | 0.5 | 0.2– 1.3 | 0.17 |
| Multifocal tumours within same breast | 3% | 2% | 1.7 | 0.3–10.6 | 0.72 |

[a]Percent of all cases, and matched controls, with factor; not restricted to discordant pairs.
[b]Late age at first birth dichotomized at <25 years and ⩾25 years and restricted to women who have given birth.

at risk to 15 years. After 15 years, the numbers at risk become too small for reliable estimation. The average annual incidence rates of second primary cancers to the contralateral breast were 5.0, 4.1 and 3.0 cases per 1000 woman-years respectively for women of age <45 years, 45–54 years and ⩾55 years at the time of diagnosis of the first primary. These differences were not significant. Figure 1 also shows the expected incidence of a second primary based on the 1971 age specific incidence rates of primary breast cancer in women in British Columbia (Cancer Register, 1975) and the age and survival distribution of the 5% sample. As expected, the differences in the expected incidence rates between age groups were large, much larger than the differences in the observed incidence rates.

The age adjusted incidence of asynchronous bilateral cancer was then determined for women with, and without, a history of breast cancer in the mother or sister (Table IV). In general the rates

**Figure 1** Incidence of a second primary in the contralateral breast in women clinically disease-free one year after the diagnosis of the first primary breast cancer by age at diagnosis of the first primary (<45 years, 45–54 years, ⩾55 years). Solid line, observed rate; broken line, expected rate. Estimated number of women at risk at 1, 5, 10 and 15 years, respectively: (a) <45 years of age, 1,374, 796, 435, 244; (b) 45–54 years of age, 2,362, 1,235, 574, 265; (c) ⩾55 years of age, 3,959, 1,827, 711, 244.

were reasonably stable over time with approximately a doubling of risk in women with a positive family history of breast cancer.

### Discussion

Incidence rates for second breast primaries have been reported to range from 3–10 cases per 1000 woman-years and from 0.3–0.9% of women with breast cancer have a second primary diagnosed within 1–6 months of the first primary (Table V and VI). Random biopsy and autopsy studies have found that the prevalence of microscopic second primary tumours is much higher, ~12% (Urban *et al.*, 1977; Berge & Ostberg, 1974).

Methodological difficulties limit the interpretation of some of these studies. Such difficulties include the use of small selected study groups, differing age distributions among the study groups with non-standardized incidence rates, and varying lengths of time between the diagnosis of the first and second primary tumours. We attempted to overcome some of these methodological problems. Our study was based on a population of over 9,000 women with over 30 years of follow-up which was analysed by a life table method.

Since the study groups are selected from patients who are referred to a tertiary care treatment centre and since the likelihood of referral could be affected by the suspicion of a second primary, the incidence of bilateral breast cancer may be over estimated. This referral bias is indicated by the high frequency of "simultaneous" second primaries (i.e. diagnosed within 1 month of the first primary) that was observed in this study as compared to other studies in the literature. We tried to minimize the

**Table IV** Age adjusted incidence of bilateral breast cancer by time interval between diagnosis and family history

| | | *Time interval between diagnosis of first and second primaries* | | | |
|---|---|---|---|---|---|
| *Family history* | | *1–5 yrs* | *5–10 yrs* | *Over 10 yrs* | *Total over 1 yr* |
| No breast cancer | Incidence[a] | 3.1 | 4.3 | 3.7 | 3.5 |
| in mother or sister | No. at risk[b] | 479 | 244 | 107 | |
| Breast cancer | Incidence[a] | 5.6 | 4.1 | 14.0 | 6.2 |
| in mother or sister | No. at risk[b] | 72 | 37 | 16 | |

[a]Age adjusted incidence rate per 1,000 woman years at risk standardized to the years at risk of the total population (i.e. 5% sample).

[b]Number of women at risk at the beginning of the time interval between diagnosis of the first and second primaries.

**Table V** Incidence of a second primary to the contralateral breast

| *Reference* | *Study population* | *Number at risk (woman years at risk)* | *Incidence (per 1000 woman years)* |
|---|---|---|---|
| *Population based studies* | | | |
| Prior & Waterhouse, 1978 | Birmingham, England 1936–1964 | 21,967 (91,233 WYR) | 4.4 |
| Mueller & Ames, 1978 | Syracuse, New York 1956–1974 | 3,558 | 8–10, to at least 15 years |
| *Hospital based studies* | | | |
| Haagensen, 1971 | Personal series 1935–1957 | 626 (6,200 WYR) | 5.8 |
| Robbins & Berg, 1964 | Memorial Hospital (N.Y.) 1940–1943 followed to 1963 | 1,458 (12,818 WYR) | 7.1, to at least 20 years |
| Fukami *et al.*, 1977 | Tokyo 1946–1975 | 3,365 (26,771 WYR) | 3.4, to at least 18 years |
| Schottenfeld & Berg, 1971 | Memorial Sloan-Kettering 1949–1962 | 9,792 (40,676 WYR) | 6.1 |
| McCredie *et al.*, 1975 | Ontario Cancer Treatment & Research Foundation 1953–1971 | 1,489, excluded subsequent metastases or recurrent disease (~50%) | ~10, to at least 20 years |
| Current study | A. Maxwell Evans Clinic 1946–1976 | over 7,800 survived at least 1 year without a second breast primary (over 45,000 WYR) | 3.8, to at least 15 years |

effect of referral biases by restricting our study to women seen at the AMEC within 1 year of diagnosis of the first primary and then by analyzing the data separately for patients with a second primary diagnosed within 1 year (synchronous cases) and after 1 year (asynchronous cases) of diagnosis of the first primary. Hence this bias should be limited to the findings for synchronous cases.

Another potential source of bias relates to the development of metastatic disease. Since the diagnosis of a second primary is clinically of limited importance in a patient with metastatic disease, one could expect a less extensive search for second primary tumours in patients with known metastatic disease. It could be argued that for this reason persons should be censored at the date of diagnosis of metastatic disease. This was not done because this date was not always recorded. We would expect that this bias would result in under-estimating the incidence rate of bilateral breast cancer because second primary tumours may be missed.

The following risk factors have generally been associated with an increased risk of bilateral breast cancer: an early age at diagnosis of the first primary (Robbins & Berg, 1964; Leis & Urban, 1978; Prior & Waterhouse, 1981; Adami *et al.*, 1981; Slack *et al.*, 1973; Hubbard, 1953); a family history of breast cancer (Armstrong & Davies, 1978; Fukami *et al.*, 1977; Leis & Urban, 1978; Hubbard, 1953; Harris *et al.*, 1978), especially breast cancer in the mother (Anderson, 1977); lobular carcinoma (Kiang *et al.*, 1980; Lewison & Neto, 1971; Robbins & Berg, 1964; Webber *et al.*, 1981); and multiple tumours within the same breast (Robbins & Berg, 1964; Leis & Urban, 1978). Conflicting results have been found for histologic grade (Robbins & Berg, 1964; Adami *et al.*, 1981), size (Robbins & Berg, 1964, Slack *et al.*, 1973) and stage (Fukami *et al.*, 1977; Robbins & Berg, 1964; Leis & Urban, 1978) of the first primary. Age at

**Table VI** Frequency of a "simultaneous" primary to the contralateral breast

| *Reference* | *Number at risk* | *Second primary* | *Definition of "simultaneous" second primary* |
|---|---|---|---|
| Prior & Waterhouse, 1978 | 21,967 | 0.4% | Diagnosed within 1 month of the first primary |
| Mueller & Ames, 1978 | 3,558 | 0.9% | Diagnosed within 6 months of the first primary |
| Haagensen, 1971 | 626 | 0.6% | Not defined |
| Robbins & Berg, 1964 | 1,458 | 0.3% | Diagnosed before removal of the first primary |
| Fukami *et al.*, 1977 | 3,365 | 0.7% | Not defined |
| Schottenfeld & Berg, 1971 | 9,792 | 0.6% | Diagnosed during treatment of the first primary |
| McCredie *et al.*, 1975 | 3,082 | 0.3% | Diagnosed within 6 months of the first primary |
| Carroll & Shields, 1955 | review of 14 studies | 1.4% | No consistent definition |
| Current study | 9,000 | 0.7% | Diagnosed within 1 month of the first primary |
| | | 1.1% | Diagnosed within 1 year of the first primary |

first birth, a well established risk factor for breast cancer, has not been associated with an increased risk of bilateral breast cancer (Adami *et al.*, 1981).

We found that a family history of breast cancer was associated with an increased risk of bilateral breast cancer, but only if the time interval between the first and second primaries exceeded one year (asynchronous cases). The reason for the absence of an association for tumours diagnosed less than one year apart (synchronous cases) in unclear. These synchronous second primaries, however, were associated with three independent factors, lobular carcinoma, absence of pathologic involvement of the axillary nodes and prior oestrogen use. The reduced risk in women with pathologic node involvement probably results from underdiagnosis of second primaries in women with metastatic disease.

Factors which influence the length of survival, such as age, stage and histologic grade, could affect the likelihood of developing a second primary and hence could be identified as important risk factors for bilateral breast cancer. We have minimized the effect of these prognostic factors by matching cases and controls on survival, at least to the diagnosis of the second primary. This effect is limited to asynchronous breast cancer for the length of survival should not influence the risk of synchronous bilateral breast cancer.

Although not statistically significant, the annual incidence of a second primary tended to decrease with later ages at diagnosis of the first primary. This has been reported in other studies. Unlike these earlier studies which compared observed numbers of second primaries with expected numbers as determined from age specific incidence rates for breast cancer, this study compared the observed incidence of second primaries at various age categories.

In conclusion, this study has found that the risk of breast cancer in the contralateral breast in women with a personal history of breast cancer is greater than the risk of breast cancer in the general population. This risk is stable over time, at least to

15 years after diagnosis of the first primary. Other risk factors for bilateral breast cancer are dependent on the length of time interval between the diagnosis of the first and second breast primaries.

We thank Dr G.M. Crawford who cared for most of these patients, the staff at the Cancer Control Agency of British Columbia for their co-operation and careful follow-up of these patients, Ms Sharon Thew and Ms Margaret Fung for abstracting the data, and Ms Lynda Jeffries for preparing the manuscript.

**References**

ADAMI, H.O., HANSEN, J., JUNG, B., LINDGREN, A. & RIMSTEN, A. (1981). Bilateral carcinoma of the breast – epidemiology and histo – *Acta. Radiol. Oncol.*, **20**, 305.

ANDERSON, D.E. (1977). Breast cancer in families. *Cancer*, **40**, 1855.

ARMSTRONG, A.E. & DAVIES, J.M. (1978). Familial breast cancer: report of a family pedigree. *Br. J. Cancer*, **37**, 294.

BERGE, T. & OSTBERG, G. (1974). Bilateral carcinoma of the female breast. *Acta. Chemis. Scand.*, **140**, 27.

BRESLOW, N.E. & DAY, N.E. (1980). *Statistical Methods in Cancer Research*, Vol. 1 – The analysis of case-control studies. *IARC Sci. Publ.*, **32**, 162.

CANCER REGISTER. (1975). Health Surveillance Registry, Ministry of Health. *Cancer in British Columbia 1969–1973. Incidence, Prevalence and Mortality.*

CARROLL, W.W. & SHIELDS, T.W. (1955). Bilateral simultaneous breast cancer. *A.M.A. Arch. Surg.*, **70**, 672.

FUKAMI, A., KASUMI, F., HORI, M. & 4 others. (1977). Bilateral primary breast cancer treated at the Cancer Institute Hospital, Tokyo. In: *Breast Cancer*, (Ed. Lewison *et al.*). New York: Alan Liss Inc., p. 525.

HAAGENSEN, C.D. (1971). *Disease of the Breast*, Saunders, Philadelphia p. 449.

HARRIS, R.E., LYNCH, H.T. & GUIRGIS, H.A. (1978). Familial breast cancer: risk to the contralateral breast. *J. Natl Cancer Inst.*, **60**, 955.

HUBBARD, T.B., Jr. (1953). Non-simultaneous bilateral carcinoma of the breast. *Surgery*, **34**, 706.

KIANG, D.T., KENNEDY, B.J. & SNOVER, D.C. (1980). Biological and histological characteristics of simultaneous bilateral breast cancer. *Lancet*, **ii**, 1105.

LEIS, H.P. & URBAN, J.A. (1978). The other breast. In: *The Breast*, (Eds. Gallagher *et al.*). St. Louis: CV Mosby Co., p. 487.

LEWISON, E.F. & NETO, A.S. (1971). Bilateral breast cancer at the John Hopkins Hospital: a discussion of the dilemma of contralateral breast cancer. *Cancer*, **28**, 1297.

McCREDIE, J.A., INCH, W.R. & ALDERSON, M. (1975). Consecutive primary carcinomas of the breast. *Cancer*, **35**, 1472.

MUELLER, C.B. & AMES, F. (1978). Bilateral carcinoma of the breast: frequency and mortality. *Can. J. Surg.*, **21**, 459.

PETO, R., PIKE, M.C., ARMITAGE, P. & 7 others. (1977). Design and analysis of randomized clinical trials requiring prolonged observation of each patient. II Analysis and Examples. *Br. J. Cancer*, **35**, 1.

PRIOR, P. & WATERHOUSE, J.A.H. (1978). Incidence of bilateral tumours in a population-based series of breast-cancer patients. I. Two approaches to an epidemiological analysis. *Br. J. Cancer*, **37**, 620.

PRIOR, P. & WATERHOUSE, J.A.H. (1981). The incidence of bilateral breast cancer: II. A proposed model for the analysis of coincidental tumours. *Br. J. Cancer*, **43**, 615.

ROBBINS, G.F. & BERG, J.W. (1964). Bilateral primary breast cancers – a prospective clinicopathological study. *Cancer*, **17**, 1501.

SCHOTTENFELD, D. & BERG, J. (1971). Incidence of multiple primary cancer, IV. Cancers of the female breast and genital organs. *J. Natl Cancer Inst.*, **46**, 161.

SLACK, N.H., BROSS, I.D.J., NEMOTO, T. & FISHER, B. (1973). Experiences with bilateral primary carcinoma of the breast. *Surg. Gynecol. Obstet.*, **136**, 433.

URBAN, J.A., PAPACHRISTOU, D. & TAYLOR, J. (1977). Bilateral breast cancer – biopsy of the opposite breast. *Cancer*, **40**, 1968.

WEBBER, B.L., HEISE, H., NEIFELD, J.P. & COSTA, J. (1981). Risk of subsequent contralateral breast carcinoma in a population of patients with in-situ breast carcinoma. *Cancer*, **47**, 2928.


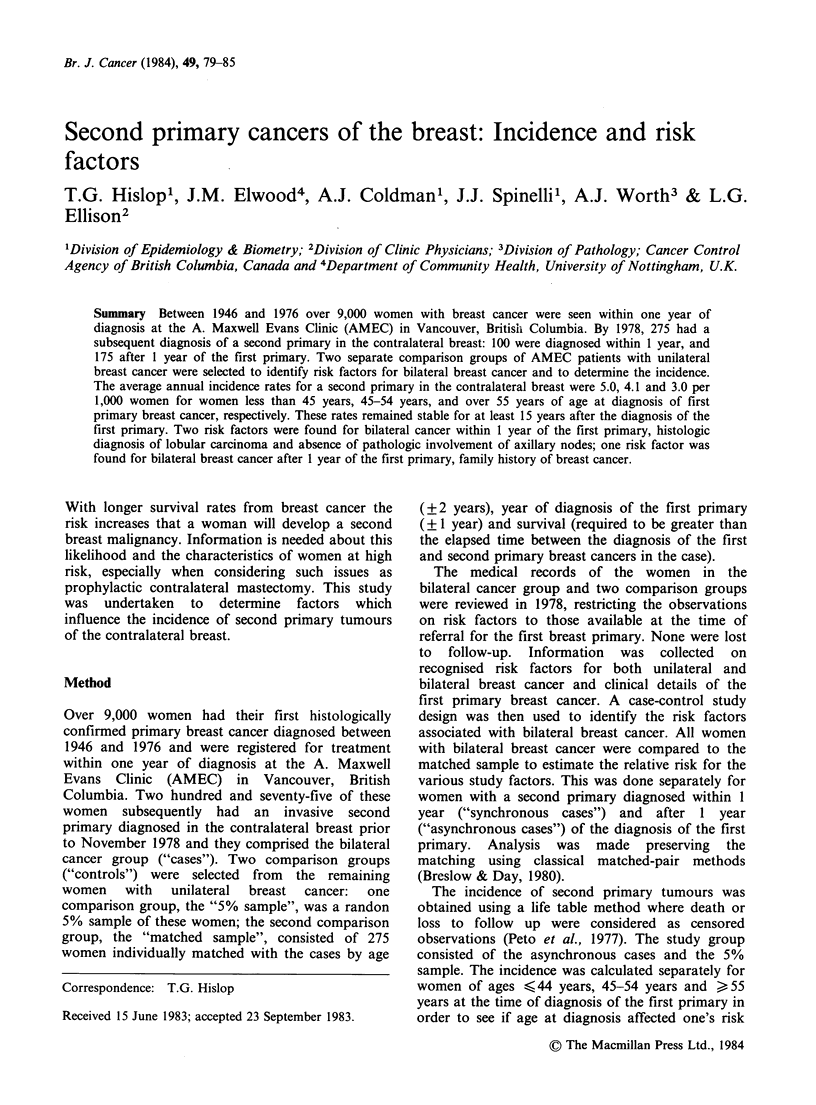

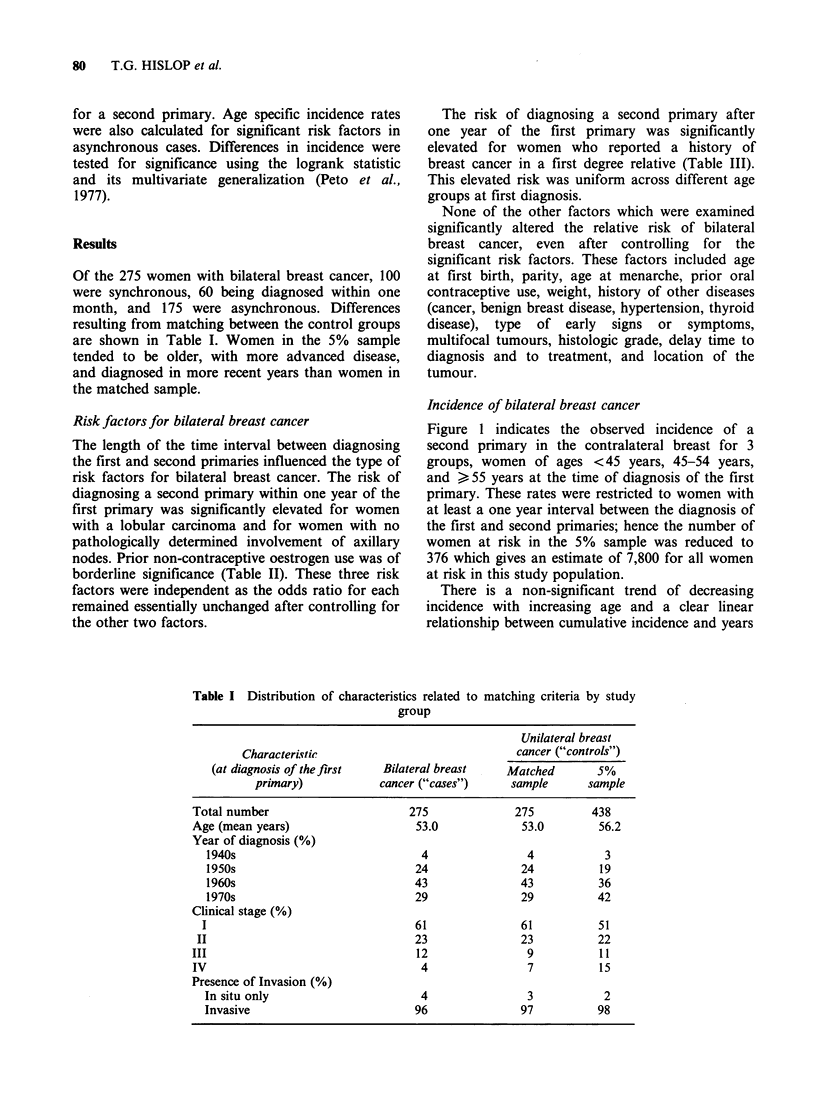

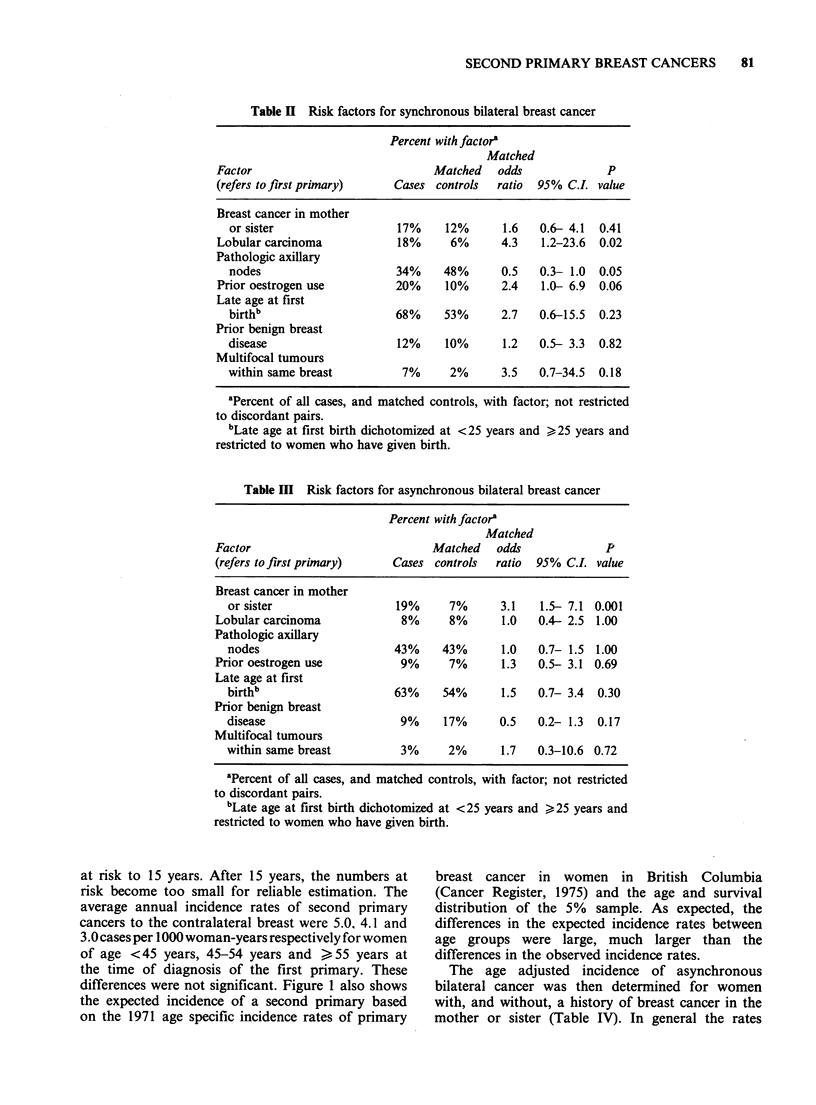

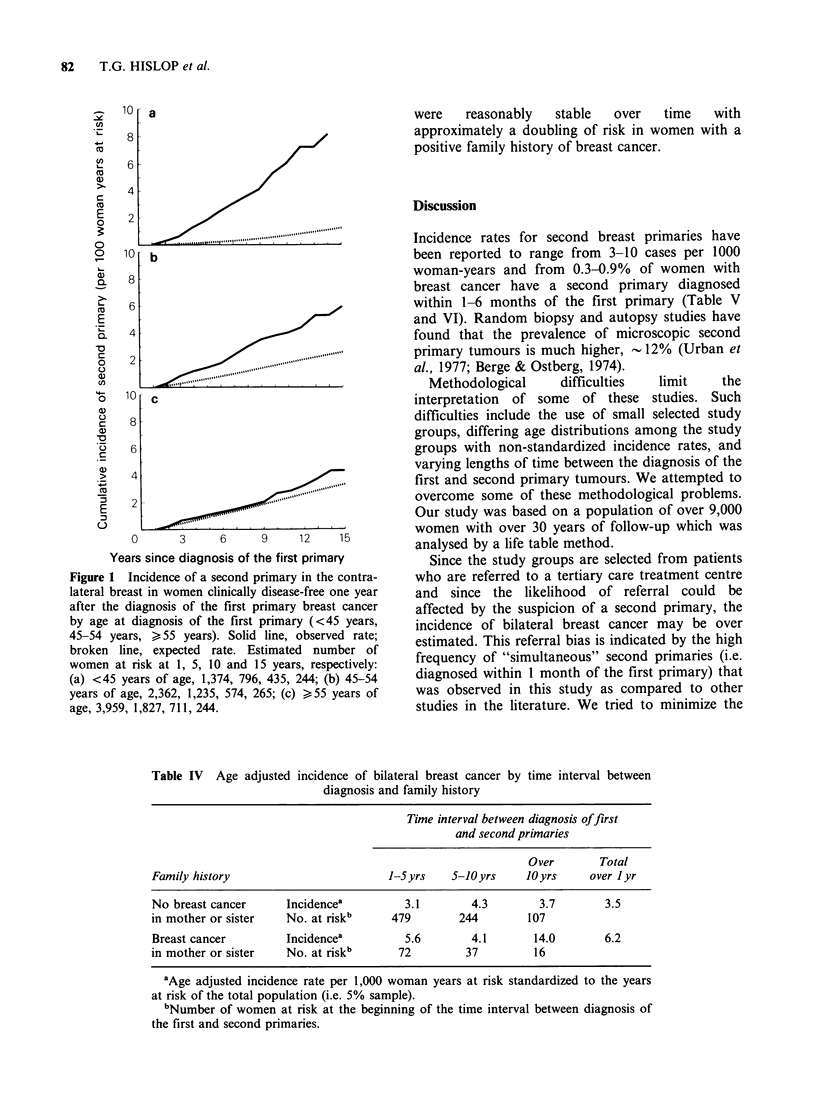

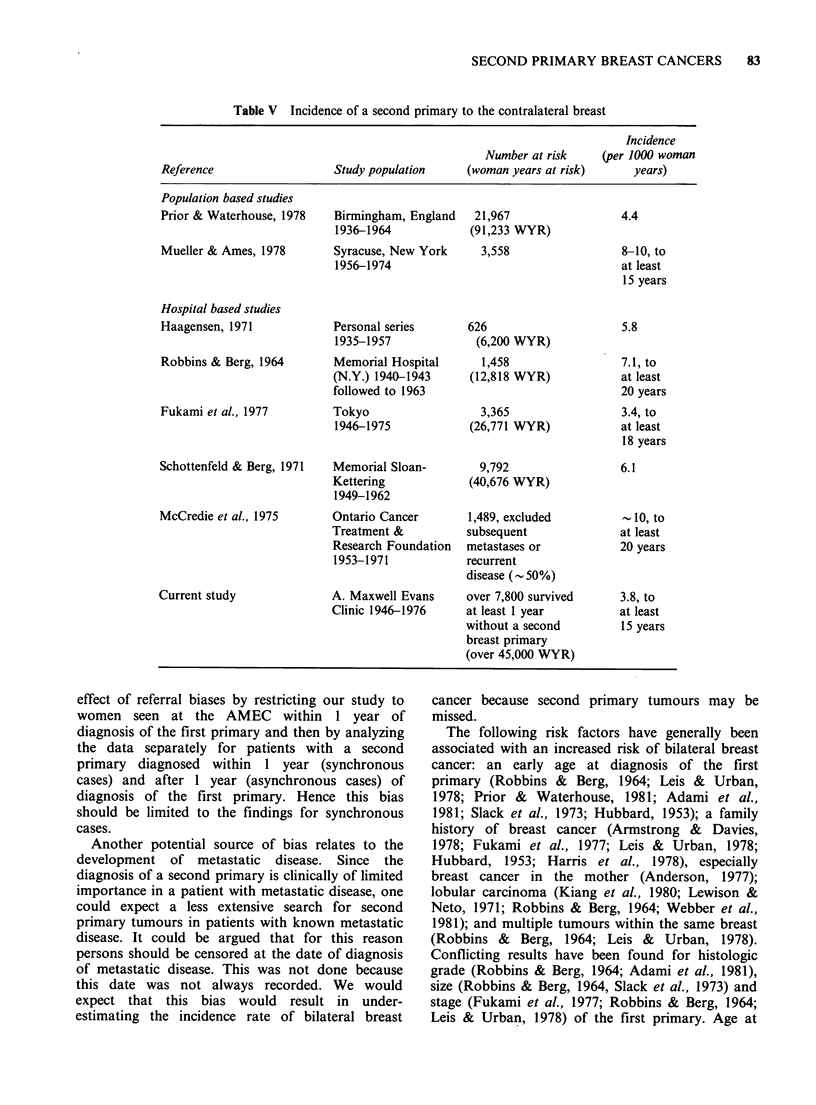

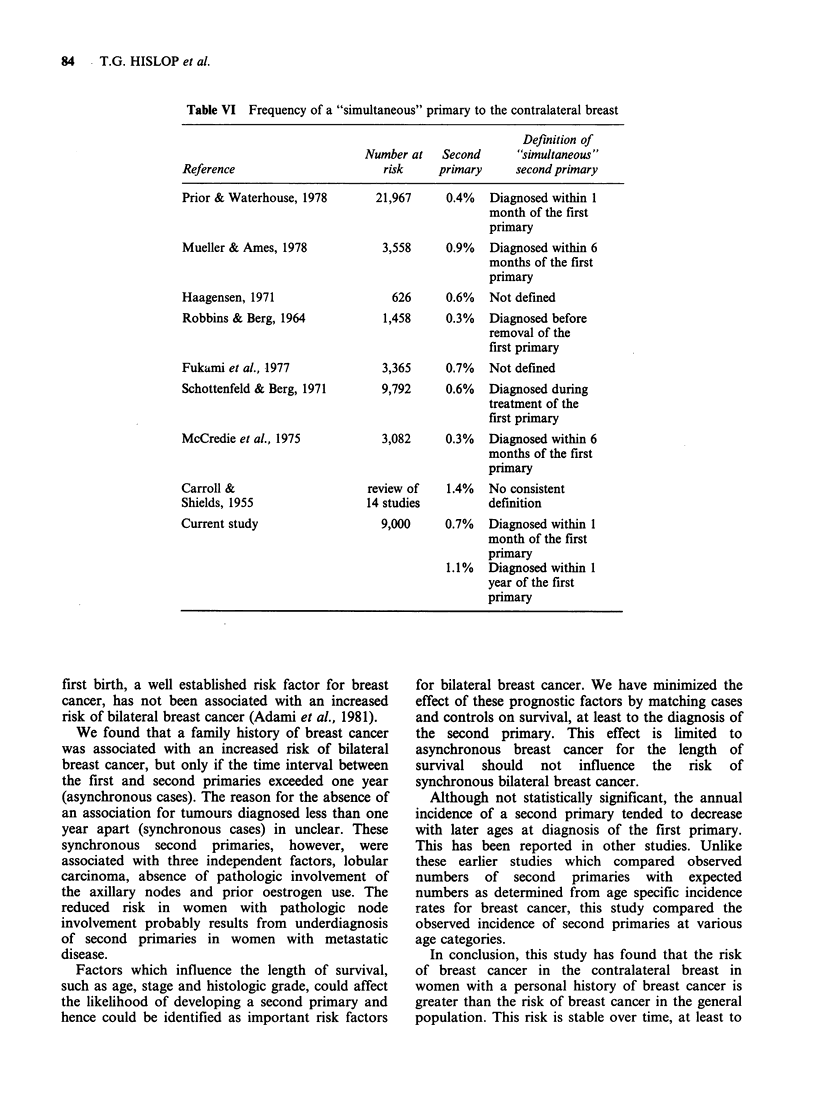

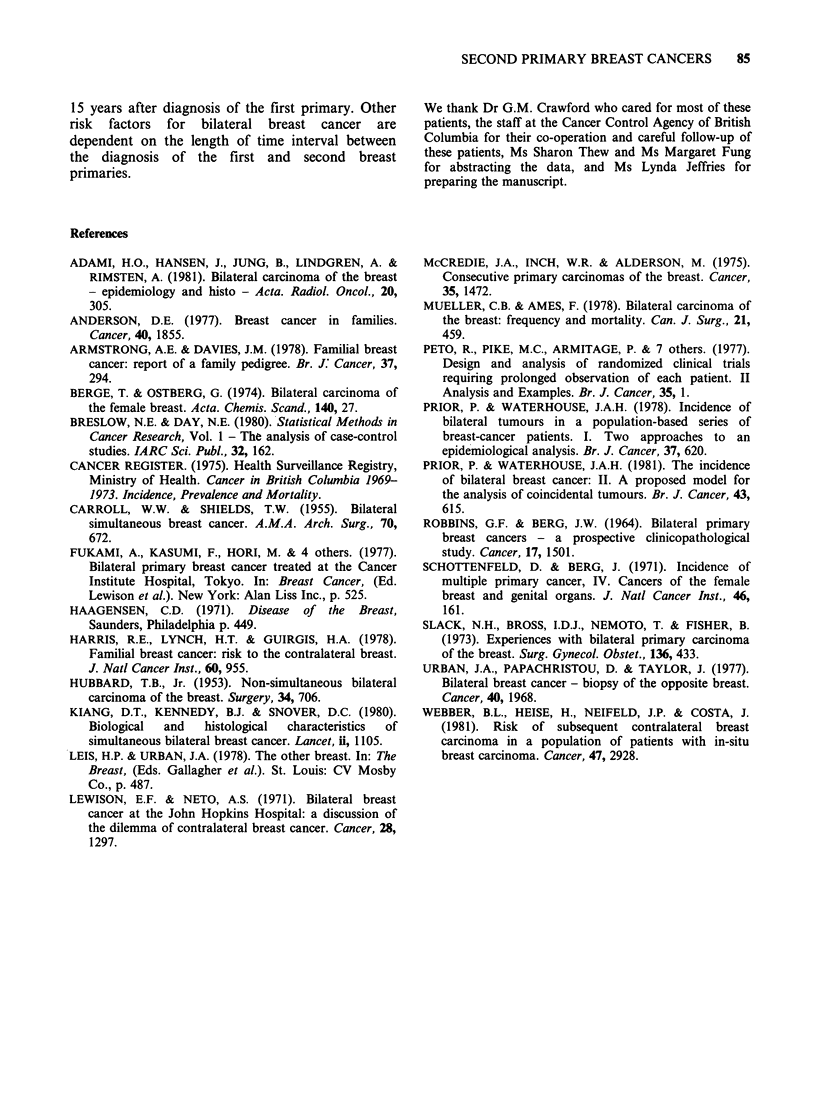


## References

[OCR_00669] Adami H. O., Hansen J., Jung B., Lindgren A., Rimsten A. (1981). Bilateral carcinoma of the breast. Epidemiology and histopathology.. Acta Radiol Oncol.

[OCR_00675] Anderson D. E. (1977). Breast cancer in families.. Cancer.

[OCR_00679] Armstrong A. E., Davies J. M. (1978). Familial breast cancer: report of a family pedigree.. Br J Cancer.

[OCR_00684] Berge T., Ostberg G. (1974). Bilateral carcinoma of the female breast.. Acta Chir Scand.

[OCR_00698] CARROLL W. W., SHIELDS T. W. (1955). Bilateral simultaneous breast cancer.. AMA Arch Surg.

[OCR_00703] Fukami A., Kasumi F., Hori M., Kuno K., Kajitani T., Sakamoto G., Sugano H. (1977). Bilateral primary breast cancer treated at the Cancer Institute Hospital, Tokyo.. Prog Clin Biol Res.

[OCR_00718] HUBBARD T. B. (1953). Nonsimultaneous bilateral carcinoma of the breast.. Surgery.

[OCR_00713] Harris R. E., Lynch H. T., Guirgis H. A. (1978). Familial breast cancer: risk to the contralateral breast.. J Natl Cancer Inst.

[OCR_00722] Kiang D. T., Kennedy B. J., Snover D. C. (1980). Biological and histological characteristics of simultaneous bilateral breast cancer.. Lancet.

[OCR_00732] Lewison E. F., Neto A. S. (1971). Bilateral breast cancer at the Johns Hopkins Hospital. A discussion of the dilemma of contralateral breast cancer.. Cancer.

[OCR_00738] McCredie J. A., Inch W. R., Alderson M. (1975). Consecutive primary carcinomas of the breast.. Cancer.

[OCR_00743] Mueller C. B., Ames F. (1978). Bilateral carcinoma of the breast: frequency and mortality.. Can J Surg.

[OCR_00754] Prior P., Waterhouse J. A. (1978). Incidence of bilateral tumours in a population-based series of breast-cancer patients. I. Two approaches to an epidemiological analysis.. Br J Cancer.

[OCR_00760] Prior P., Waterhouse J. A. (1981). The incidence of bilateral breast cancer: II. A proposed model for the analysis of coincidental tumours.. Br J Cancer.

[OCR_00766] ROBBINS G. F., BERG J. W. (1964). BILATERAL PRIMARY BREAST CANCER; A PROSPECTIVE CLINICOPATHOLOGICAL STUDY.. Cancer.

[OCR_00771] Schottenfeld D., Berg J. (1971). Incidence of miltiple primary cancers. IV. Cancers of the female breast and genital organs.. J Natl Cancer Inst.

[OCR_00777] Slack N. H., Bross I. D., Nemoto T., Fisher B. (1973). Experiences with bilateral primary carcinoma of the breast.. Surg Gynecol Obstet.

[OCR_00782] Urban J. A., Papachristou D., Taylor J. (1977). Bilateral breast cancer: biopsy of the opposite breast.. Cancer.

[OCR_00787] Webber B. L., Heise H., Neifeld J. P., Costa J. (1981). Risk of subsequent contralateral breast carcinoma in a population of patients with in-situ breast carcinoma.. Cancer.

